# 16S Ribosomal RNA Gene PCR and Sequencing for Pediatric Infection Diagnosis, United States, 2020–2023

**DOI:** 10.3201/eid3113.241101

**Published:** 2025-05

**Authors:** Guyu Li, Christopher A. Reis, Rebecca M. Kruc, Ziyuan Zhang, Nicholas T. Streck, Elizabeth H. Ristagno, Jay Mandrekar, Matthew J. Wolf, James T. Gaensbauer, Robin Patel

**Affiliations:** Mayo Clinic, Rochester, Minnesota, USA (G. Li, C.A. Reis, R.M. Kruc, N.T. Streck, E.H. Ristagno, J. Mandrekar, M.J. Wolf, J.T. Gaensbauer, R. Patel); Harvard T.H. Chan School of Public Health, Boston, Massachusetts, USA (Z. Zhang)

**Keywords:** 16S ribosomal RNA, PCR, sequencing, bacteria, bacterial infections, pediatrics, United States

## Abstract

Gene PCR and sequencing using 16S ribosomal RNA (rRNA) can help diagnose challenging bacterial infections. Data on the optimal clinical settings for this type of testing are limited. We performed a retrospective study at Mayo Clinic, Rochester, Minnesota, USA, with typically sterile specimens from children that underwent 16S rRNA PCR testing during September 2020–December 2023. Of 162 tests performed on 124 patients, 20% were positive; 58% of positive samples were from culture-negative specimens. Fluid specimens were >3 times as likely to test positive as tissue specimens (odds ratio 3.07 [95% CI 1.32–7.11]; p = 0.007), and pleural fluid demonstrated the highest positivity rate (50%). Of 33 positive results, 4 (12%) specimens qualified for reporting to the state health department for communicable diseases. Those single-laboratory findings demonstrate that the highest positivity rate of 16S rRNA PCR and sequencing is pleural fluid, although many specimen types tested positive.

Gene PCR using 16S ribosomal RNA (rRNA) followed by sequencing can identify bacteria in normally sterile body tissues and fluids (*1*,*2*). This method may serve as a diagnostic tool in complex bacterial infections, particularly when conventional tests fail to identify pathogens (*3*,*4*). The clinical use of 16S rRNA PCR and sequencing has been shown to yield concordant results with bacterial cultures (when positive), to enhance detection of fastidious bacteria, and to assist in antimicrobial drug stewardship (*4*–*8*). However, the diagnostic yield of 16S rRNA PCR and sequencing from various specimen sources has been variable in published studies (*4*,*6*,*9*–*11*); diagnostic yield may vary on the basis of patient and specimen characteristics. Data on optimal clinical settings and specimen selection for this testing are limited, particularly in pediatrics (*9*,*12*).

Mayo Clinic (Rochester, MN, USA) began offering 16S rRNA PCR and sequencing clinically in 2017; the sequencing initially involving Sanger sequencing alone (*4*). Then, in 2019, to increase positivity rates and to decatenate mixed sequences because of 16S rRNA gene copy variants or polymicrobial infections, next-generation sequencing (NGS) was substituted for or added to Sanger sequencing of the PCR-amplified 16S rRNA gene when needed (*13*). This study reviews Mayo Clinic’s clinical experience with 16S rRNA PCR and sequencing of specimens from children to identify clinical syndromes where this testing is useful and to optimize specimen choice.

## Methods

### Study Design

We performed a retrospective study involving specimens collected from Mayo Clinic patients 0–18 years of age whose normally sterile tissue or fluid specimens underwent 16S rRNA PCR and sequencing during September 2020–December 2023. We identified patients and 16S rRNA PCR and sequencing results by using the clinical microbiology laboratory database and collected demographic, clinical, and microbiologic data from the electronic medical record. If a patient had specimens collected from the same source during different encounters, we included only specimens from the first encounter. In routine clinical practice, holding a specimen in the clinical microbiology laboratory for 14 days for potential 16S rRNA PCR and sequencing, if clinically needed, was offered as an option. This study was approved by the Mayo Clinic Institutional Review Board (protocol no. 20–012373).

### Definitions

Immunocompromised hosts included patients with malignancies undergoing chemotherapy, those who had undergone solid organ or hematopoietic stem cell transplantation, and those receiving high-dose steroids (pulse dose steroids 20 mg/d for >14 days, or dexamethasone for >10 days) or other immunosuppressive agents. We defined intensive care unit (ICU) admission as receiving medical care in the neonatal, pediatric, or cardiovascular ICU at the time of specimen collection.

We categorized cerebrospinal fluid, ovarian fluid, pericardial fluid, peritoneal fluid, pleural fluid, subdural fluid, synovial fluid, and vitreous fluid as fluid specimens and other specimens (e.g., bone) as tissue specimens. We collected the results of conventional testing, which included Gram stain, bacterial culture, BioFire Meningitis and Encephalitis (ME) panel (bioMérieux, https://www.biomerieux.com), and *Kingella kingae* PCR if clinically performed on specimens collected at the same time and from the same site as specimens for 16S rRNA PCR and sequencing. We calculated the turnaround time as the interval from specimen collection to result finalization. We defined prior antibacterial therapy as any antimicrobial drugs administered within 24 hours before the test order for 16S rRNA PCR and sequencing.

### Specimen Processing

We performed specimen processing and bacterial culture in the Clinical Bacteriology Laboratories of the Division of Clinical Microbiology at Mayo Clinic. We identified isolated bacteria by using conventional biochemical methods or matrix-assisted laser desorption/ionization time-of-flight mass spectrometry. Details of the 16S rRNA PCR and sequencing procedure have been described previously (*13*). In brief, the assay involved an up-front real-time PCR assay, reported as negative or submitted to Sanger or NGS on the basis of cycle threshold (Ct) value. Specimens with Ct values <32 cycles underwent bidirectional Sanger sequencing by using an Applied Biosystems 3500xL Genetic Analyzer (Thermo Fisher Scientific, https://www.thermofisher.com). We sent specimens with Ct values of 32–34 or <32 with Sanger sequencing that yielded an uninterpretable result to NGS by using an Illumina MiSeq System (Illumina, https://www.illumina.com) with a 500-cycle (2 × 250 paired-end read) v2 nano kit. We reported specimens with Ct values >34 as negative, except if we observed a well-defined melting temperature peak (>0.4), in which case we sent them to NGS. We used Pathogenomix (https://www.pathogenomix.com) for quality control processes and the Pathogenomix PRIME database for sequence analysis. The Pathogenomix Prime database contains 48,139 curated 16S rRNA gene sequences. The processor filters low-quality reads (Q<30) and clusters sequences on the basis of >210-bp length, >100 copies, and 0% variation.

### Statistical Analysis

We compared characteristics between positive and negative tests by using a 2-sample *t*-test for continuous variables. For categorical variables with >5 observations, we calculated odds ratios (ORs) and 95% CIs by using unconditional maximum likelihood estimation; we obtained p values by using χ^2^ tests. For categorical variables with <5 observations, we calculated ORs and 95% CIs by using conditional maximum likelihood estimation and obtained p values were by using Fisher exact tests. We considered a 2-tailed p value <0.05 statistically significant.

## Results

### Patients

A total of 124 pediatric patients with 162 tests from typically sterile sources were included (Figure). At sampling, 20% (n = 24) of patients were identified as immunocompromised hosts, and 37% (n = 46) of patients were in ICUs (Table 1). The most common suspected clinical manifestations were meningoencephalitis, musculoskeletal infection, and pleural effusion.

**Figure:**
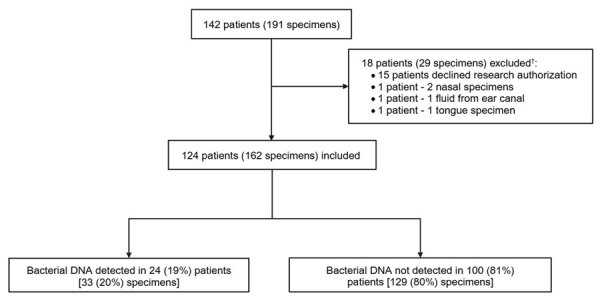
Specimen flowchart from a study of 16S ribosomal RNA gene PCR and sequencing for pediatric infection diagnosis, Mayo Clinic, Rochester, Minnesota, USA, 2020–2023. Specimens from tongue, ear canal, and nose were excluded.

**Table 1:** Patient characteristics from study of 16S ribosomal RNA gene PCR and sequencing for pediatric infection diagnosis, Mayo Clinic, Rochester, Minnesota, USA, 2020–2023*

Patient characteristics	Value, n = 124 patients
Median age, y (IQR)	9.6 (2.2–15.0)
Sex	
F	58 (47)
M	66 (53)
Immunocompromised host†	24 (20)
Intensive care unit admission	46 (37)
Suspected clinical syndrome	
Meningoencephalitis	38 (31)
Musculoskeletal infection: septic arthritis, osteomyelitis	33 (27)
Pleural effusion	10 (8)
Surgical wound infection, including hardware infection	10 (8)
Lymphadenopathy	5 (4)
Bone mass	4 (3)
Intracranial abscess/fluid collection	2 (2)
Pericardial effusion	4 (3)
Pulmonary nodules	4 (3)
Traumatic wound infection	4 (3)
Intraabdominal abscess/fluid collection	4 (3)
Endocarditis	2 (2)
Splenic mass	1 (1)
Infected pseudoaneurysms	1 (1)
Mediastinitis	1 (1)
Retinal detachment	1 (1)
Suggested by pediatric infectious diseases team	56 (45)

*Values are no. (%) patients except as indicated. IQR, interquartile range. †Immunocompromised hosts include those with history of solid organ transplant, history of hematopoietic stem cell transplant, active chemotherapy, or receiving an immunosuppressive agent.

### 16S rRNA PCR and Sequencing Results

The mean turnaround time for positive 16S rRNA PCR and sequencing tests was 8 days (3.2–12.8 days), whereas for negative tests it was 3 days (0–6.7 days) (Table 2). A total of 84 (50%) specimens were collected from patients who received antimicrobial drugs within 24 hours before sampling, which was associated with a higher likelihood of positive results (p = 0.001).

**Table 2:** Specimen characteristics from study of 16S ribosomal RNA gene PCR and sequencing for pediatric infection diagnosis, Mayo Clinic, Rochester, Minnesota, USA, 2020–2023*

Specimen characteristics	Positive 16S rRNA PCR and sequencing, n = 33	Negative 16S rRNA PCR and sequencing, n = 129	OR (95% CI)	p value
Turnaround time, d, mean ± SD	8.0 ± 4.8	3.0 ± 3.7	NA	<0.0001
Received antimicrobial drugs within 24 h	28 (85)	56 (43)	7.30 (2.65–20.11)	0.001
Specimen hold ordered before testing	3 (9)	14 (11)	0.82 (0.22–3.04)	1.000
Specimen type				
Fluid	24 (73)	60 (47)	3.07 (1.32–7.11)	0.007
Tissue	9 (27)	69 (54)	0.33 (0.14–0.76)	0.007
Specimen source†				
Cerebrospinal fluid	5 (15)	33 (26)	NA	NA
Bone tissue	4 (12)	24 (19)	NA	NA
Deep soft tissue	0	22 (17)	NA	NA
Synovial fluid	9 (27)	12 (9)	NA	NA
Pleural fluid	5 (15)	5 (4)	NA	NA
Synovial tissue	2 (6)	7 (5)	NA	NA
Lymph node	0	5 (4)	NA	NA
Subdural fluid	3 (9)	1 (1)	NA	NA
Pericardial fluid	0	4 (3)	NA	NA
Peri-implant tissue	2 (6)	2 (2)	NA	NA
Peritoneal fluid	1 (3)	2 (2)	NA	NA
Lung parenchyma	0	3 (2)	NA	NA
Pacemaker pocket tissue	0	3 (2)	NA	NA
Vitreous fluid	1 (3)	1 (1)	NA	NA
Brain tissue	0	2 (2)	NA	NA
Ovarian fluid	0	1 (1)	NA	NA
Heart valve tissue	1 (3)	0	NA	NA
Spleen tissue	0	1 (1)	NA	NA
Vascular tissue	0	1 (1)	NA	NA

*Values are no. (%) tests except as indicated. NA, not applicable; OR, odds ratio. †Statistical analysis was not performed because of the limited sample size.

The overall 16S rRNA PCR and sequencing positivity rate was 20% among all 162 specimens collected from 124 patients (Figure). Fluid specimens were 3-fold more likely to test positive compared with tissue specimens (OR 3.07 [95% CI 1.32–7.11]; p = 0.007) (Table 2). The most frequent specimen sources were cerebrospinal fluid, bone tissue, deep soft tissue, synovial fluid, and pleural fluid. Among those, specimens with high positivity rates included pleural fluid (50%, n = 5) and synovial fluid (43%, n = 9); there were no positive results from deep soft tissue specimens.

Among the 33 positive tests, 12 (36%) tests were polymicrobial detections. The most common single bacteria identified was *Staphylococcus aureus* complex in 4 (12%) positive tests, followed by *Kingella kingae* in 3 (9%) positive tests (all synovial fluid); other bacteria each accounted for 3%–9% of positive tests from various sources (Table 3). We recorded details of test results and clinical diagnoses for 24 patients with positive 16S rRNA PCR and sequencing results (Appendix Table).

**Table 3:** Microorganisms detected by 16S rRNA PCR and sequencing and associated specimen sources from a study of 16S ribosomal RNA gene PCR and sequencing for pediatric infection diagnosis, Mayo Clinic, Rochester, Minnesota, USA, 2020–2023

Identified bacteria	No. (%) positive results, n = 33	Specimen source (no. tests)
Polymicrobial	12 (36)	Bone tissue (4*), subdural fluid (3*), cerebrospinal fluid (1), peri-implant tissue (1), vitreous fluid (1*), synovial tissue (1), peritoneal fluid (1*)
*Staphylococcus aureus* complex	4 (12)	Synovial fluid (4)
*Kingella kingae*	3 (9)	Synovial fluid (3*)
*Streptococcus mitis* group	3 (9)	Pleural fluid (1), pleural fluid (2*)
*Fusobacterium naviforme/nucleatum*	2 (6)	Synovial fluid (1*), CSF (1*)
*Streptococcus intermedius*	2 (6)	Cerebrospinal fluid (2)
*Cardiobacterium hominis*	1 (3)	Peri-implant tissue (1*)
*Enterococcus faecalis*	1 (3)	Heart valve tissue (1)
*Fusobacterium necrophorum*	1 (3)	Pleural fluid (1)
*Pseudomonas aeruginosa*	1 (3)	Synovial tissue (1*)
*Staphylococcus epidermidis*	1 (3)	Cerebrospinal fluid (1)
*Streptococcus dysgalactiae*	1 (3)	Synovial fluid (1*)
*Streptococcus pyogenes*	1 (3)	Pleural fluid (1*)

*Specimens with negative bacterial cultures from the same specimen source.

### Comparison to Conventional Tests

Among 152 specimens tested with both Gram stain and 16S rRNA PCR and sequencing, 21% (n = 7) of positive specimens had a corresponding positive Gram stain, whereas none of the negative tests were associated with a positive Gram stain. Patients with positive Gram stains had a higher likelihood of positive 16S rRNA PCR and sequencing results compared with patients with negative Gram stains (p<0.0001) (Table 4).

**Table 4:** Comparison of conventional tests and 16S rRNA PCR and sequencing results from study of 16S ribosomal RNA gene PCR and sequencing for pediatric infection diagnosis, Mayo Clinic, Rochester, Minnesota, USA, 2020–2023*

Conventional tests characteristics	16S rRNA PCR and sequencing results		p value
	Positive	Negative	
Gram stain			
Positive	7	0	<0.0001
Negative	26	119	
Bacterial culture			
Positive	14	9	<0.0001
Negative	19	119	
BioFire Meningitis/Encephalitis panel, cerebrospinal fluid only			
Positive	0	2†	1.000
Negative	2	19	
Synovial fluid *Kingella kingae* PCR, when clinically ordered			
Positive	3	0	0.143
Negative	1	3	

*Specimens were collected from same specimen source. †One panel was positive for parechovirus and another was positive for human herpesvirus 6.

Of the 161 specimens tested with both bacterial cultures and 16S rRNA PCR and sequencing, 133 (83%) specimens demonstrated concordant results between the 2 methods: 14 (9%) specimens were positive after both tests and 119 (74%) specimens were negative after both tests. In addition, 19 specimens with negative bacterial cultures were positive by 16S rRNA PCR and sequencing: polymicrobial infections (n = 9), *K. kingae* (n = 3), *Fusobacterium naviforme/nucleatum* (n = 2), *Streptococcus mitis* group (n = 2), *Cardiobacterium hominis* (n = 1), *Pseudomonas aeruginosa* (n = 1), and *Streptococcus pyogenes* (n = 1).

Nine 16S rRNA PCR and sequencing tests were negative despite positive cultures: 4 positive results for *Cutibacterium acnes* from bacterial cultures in periimplant and bone tissue, 1 positive result for *Staphylococcus capitis* from bacterial culture in a bone tissue specimen, and 5 cases of suspected culture contamination. The contamination cases involved isolations of *Staphylococcus epidermidis* from pleural fluid (n = 1), deep soft tissue (n = 1), and lymph node tissue (n = 1) and *Niallia circulans* group from bone (n = 1) and deep soft tissue (n = 1).

Of the 23 specimens tested with both the BioFire ME panel and 16S rRNA PCR and sequencing, 2 were negative by the panel with positive 16S rRNA PCR and sequencing results (*S. epidermidis* and *F. naviforme/nucleatum*). The *S. epidermidis* case was considered a contaminant. No bacterial pathogens were identified by the BioFire ME panel that were not also detected by 16S rRNA PCR and sequencing.

Of the 7 synovial fluid specimens tested with both *K. kingae* PCR and 16S rRNA PCR and sequencing, 86% (n = 6) of specimens showed concordant positive or negative results. 16S rRNA PCR and sequencing detected *K. kingae* in 1 synovial fluid specimen that tested negative with synovial fluid *K. kingae* PCR.

### Multiple Tests on the Same Specimen Type

At least 2 16S rRNA PCR and sequencing tests were ordered for 22 patients on the same specimen source during the same procedure (Table 5), mostly bone tissue, deep soft tissue, and synovial fluid. All tests yielded concordant results, either negative or positive.

**Table 5:** Characteristics of multiple 16S rRNA PCR and sequencing tests ordered on the same patient from the same specimen source during the same procedure from study of 16S ribosomal RNA gene PCR and sequencing for pediatric infection diagnosis, Mayo Clinic, Rochester, Minnesota, USA, 2020–2023*

Characteristics	Bone tissue, n = 7	Deep soft tissue, n = 6	Synovial fluid, n = 4	Cerebrospinal fluid, n = 2	Brain tissue, n = 1	Pacemaker pocket tissue, n = 1	Subdural fluid, n = 1
Frequency of tests ordered							
Two	4 (57)	3 (50)	3 (75)	2 (100)	1 (100)	1 (100)	0
Three	2 (29)	3 (50)	1 (25)	0	0	0	1 (100)
Four	1 (14)	0	0	0	0	0	0
Assay results							
Concordant negative	6 (86)	6 (100)	3 (75)	1 (50)	1 (100)	1 (100)	0
Concordant positive	1 (14)	0	1 (25)	1 (50)	0	0	1 (100)
Discordant	0	0	0	0	0	0	0

*Values are no. (%) patients.

### Specimen Hold Strategy

Clinicians placed a request to hold a specimen for potential future 16S rRNA PCR and sequencing testing on 17 specimens (Table 2). Over the ensuing clinical course, because of positive Gram stains and negative bacterial cultures after 24–48 hours of incubation, 16S rRNA PCR and sequencing tests were performed on the saved specimens. Of those, 3 tests had positive 16S rRNA PCR and sequencing results, including identification of *S. mitis* group in 2 pleural fluid specimens and *S. dysgalactiae* in 1 synovial fluid specimen.

## Discussion

In this study, we conducted a 3-year retrospective evaluation of the diagnostic yield of 16S rRNA PCR and sequencing in children by using various specimen types. We were unable to find many other published studies exploring the application of 16S rRNA PCR and sequencing in pediatric patients. The overall test positivity rate we found was 20%, consistent with previous studies in pediatric patients, which reported positivity rates ranging from 14% to 23% (*6*,*9*,*10*,*14*). Initiation of empiric therapy within 24 hours before sampling did not negatively affect positivity rates, consistent with findings from studies in adults and children (*4*,*9*,*11*). 

Subgroup analysis revealed that 16S rRNA PCR and sequencing had a higher positivity rate in fluid compared with tissue specimens, especially in pleural fluid, which provided additional diagnostic value for pathogens such as *S. mitis* group and *S. pyogenes*. Despite the limited pediatric sample size, our findings are consistent with prior studies indicating that pleural fluid yields a high positivity rate (*10*,*14*,*15*).

A potential limitation of our study is that bronchoalveolar lavage (BAL) fluid was not tested; in prior studies, BAL fluid has been reported as a common specimen source for 16S rRNA PCR and sequencing testing. However, despite high positivity rates in BAL fluids, the clinical relevance of those findings has been questionable (*6*,*9*,*16*), possibly because BAL fluid is not sterile. In contrast, sample dilution during bronchoscopy can increase the likelihood of false-negative results.

The yield of 16S rRNA PCR and sequencing in bone and joint infection has varied in previous research, ranging from 21% to 32% (*17*–*19*). Bone tissue and synovial fluids or tissues were the most common sources in this study. Compared with the single *K. kingae* PCR test on synovial fluid used at the Mayo Clinic, the 16S rRNA PCR and sequencing offered additional diagnostic value in only 1 of 7 cases. Given the shorter turnaround time of the *K. kingae* PCR test, a single PCR test remains the optimal first-line test for suspected bone and joint infections in toddlers. This target is also available on the BioFire joint infection (JI) panel (*20*). The BioFire JI panel has been used for rapid diagnosis of pediatric septic arthritis, offering a fast turnaround, and sensitive and specific detection of on-panel microorganisms and select antimicrobial resistance genes (*20*,*21*). Compared with the BioFire JI panel, 16S rRNA PCR and sequencing demonstrated higher sensitivity in periprosthetic JI (PJI) because the BioFire JI panel does not include *S. epidermidis*, a common cause of PJI (*22*–*24*).

We found discrepancies in *C. acnes* testing, in which cultures were positive but 16S rRNA PCR and sequencing was negative (4 peri-implant and bone tissue specimens). Those discrepancies are likely because of the limited ability to report low abundance *C. acnes* from 16S rRNA PCR and sequencing because of its frequent presence in background sequences, as published previously (*25*,*26*).

Of the 23 BioFire ME panels performed, 19 had concordant negative results by 16S rRNA PCR and sequencing, in keeping with other studies’ findings (*4*,*27*). 16S rRNA PCR and sequencing uniquely identified *F. naviforme/nucleatum*, which is not included in the ME panel (*28*). Two cases were negative by 16S rRNA PCR and sequencing but positive for viruses by the BioFire ME panel; this is expected because 16S rRNA PCR and sequencing targets bacterial DNA, while the BioFire ME panel includes viral targets. Turnaround time is a key factor to consider. Our findings underscore the value of using the BioFire ME panel ahead of 16S rRNA PCR and sequencing, proceeding to 16S rRNA PCR and sequencing when the BioFire ME panel is negative (*29*).

In this study, multiple 16S rRNA PCR and sequencing tests performed on specimens from the same specimen source collected during the same procedure resulted in no discordant results. Assessment of the clinical value of performing multiple 16S rRNA PCR and sequencing tests has been limited. A multicenter study on adult PJI showed that collecting 5 perioperative samples per patient for culture and 16S rRNA PCR and sequencing showed a lack of sensitivty of the latter in the diagnosis of PJI (*30*). Another report indicated that testing multiple samples per patient may help rule out potential contaminating microorganisms (*31*). Our findings indicate a single 16S rRNA PCR and sequencing test on 1 specimen, collected along with at >2 specimens for bacterial culture during the same procedure, may be adequate.

This study also explored the role of collecting and holding a specimen for future testing if clinically indicated. Positive detections were found in 3 cases managed with this strategy. We conceive that use of this diagnostic pathway could optimize testing resource use. Further research with larger sample sizes is necessary to determine the clinical syndromes and specimen sources that would benefit from delayed or reflexive testing.

The use of 16S rRNA PCR and sequencing in clinical practice has implications for public health, including enhanced detection of bacteria that may be notifiable infectious diseases. Clinical laboratories should establish protocols for reporting detected pathogens to public health authorities, and public health laboratories should define which molecularly detected species are reportable from which specimen types. As demonstrated in this study, *K. kingae*, often missed by conventional cultures, is readily detected by 16S rRNA PCR and sequencing. Clinical use of this assay can provide data useful for identifying outbreaks and informing timely public health interventions (*32*).

The first limitation of this study is that the small sample size limits statistical power. Second, the study was conducted at a single institution, limiting generalizability. Finally, subgroup analysis of suspected clinical syndromes and outcomes was not performed. Future studies with larger sample sizes, specimens collected from multiple sites, comprehensive clinical outcomes recorded, and adjustments for potential confounders are warranted.

In conclusion, this study demonstrates that 16S rRNA PCR and sequencing yields the highest positivity rate in fluid specimens, particularly pleural and synovial fluids from children. A strategy of collecting specimens for future testing, if clinically indicated, is described as a diagnostic stewardship tool. Further research should focus on optimizing use of the described testing use in conjunction with other testing, while considering overall turnaround time. Implementation research is needed to evaluate the effect of 16S rRNA PCR and sequencing on patient outcomes.

Appendix Additional information about study of 16S ribosomal RNA gene PCR and sequencing for pediatric infection diagnosis, USA, 2020–2023.
