# Supplementary material for: 16S Ribosomal RNA Gene PCR and Sequencing for Pediatric Infection Diagnosis, United States, 2020–2023
Source: Emerg Infect Dis. 2025 May;31(Suppl 1):S129–36. doi: 10.3201/eid3113.241101 (PMC12078542; doi:10.3201/eid3113.241101)
Supplement: Appendix — Additional information about study of 16S ribosomal RNA gene PCR and sequencing for pediatric infection diagnosis, USA, 2020–2023. [file 24-1101-Techapp-s1.pdf]

Article DOI: https://doi.org/10.3201/eid3113.241101

EID cannot ensure accessibility for supplementary materials supplied by authors. Readers who have difficulty accessing supplementary content should contact the authors for assistance.

16S Ribosomal RNA Gene PCR and Sequencing for Pediatric Infection Diagnosis, United States, 2020–2023

Appendix.

Appendix Table. Tests results and clinical diagnosis in 24 patients with positive 16S rRNA gene PCR/ sequencing results*

No.	Age	Suspected clinical syndrome	Specimen source	16S rRNA gene PCR/sequencing test results	Conventional tests from same specimen source	Clinical diagnosis
1	6 mo	Endocarditis	Heart valve	*Enterococcus faecalis*	- Gram stain negative, all specimens.<br>- Bacterial culture grew *E. faecalis* from same specimen.	Endocarditis
2	10 yo	Endocarditis	Peri-implant tissue	*Cardiobacterium hominis*	- Gram stain and bacterial cultures negative, all specimens.	Endocarditis
3	18 yo	Hardware infection	Synovial tissue	*Pseudomonas aeruginosa*	- Gram stain and bacterial cultures negative, all specimens.	Periprosthetic joint infection
4	15 yo	Hardware infection	Synovial fluid	*Staphylococcus aureus* complex	- Gram stain negative, all specimens.<br>- Bacterial culture grew *S. aureus* complex from same specimen.	Orthopedic hardware-related osteomyelitis
5	3 yo	Intraabdominal abscess/ fluid collection	Peritoneal fluid	*Alistipes onderdonkii, Bacteroides fragilis, Dysgonomonas* species *Phocaeicola vulgatus, Clostridium/Eubacterium* species, *Intestinimonas butyriciproducens, Alistipes finegoldii, Sutterella* species, *Campylobacter* species, *Bacteroides* species, *Enterobacteriaceae, Alloprevotella* species, *Dialister invisus*	- Gram stains showed Gram-positive cocci, Gram-negative bacilli, Gram-positive bacilli.<br>- Bacterial cultures grew *Enterococcus faecium, Escherichia coli, Dysgonomonas mossii/oryzarvi* from same specimens.	Polymicrobial infection due to perforated appendicitis.
6	14 yo	Intracranial abscess/ fluid collection	Subdural fluid<br>Subdural fluid<br>Subdural fluid	*Porphyromonas endodontalis, Segatella oris*<br>*P. endodontalis, S. oris*<br>*P. endodontalis, S. oris*	- Gram stain and bacterial cultures negative, all specimens.	Subdural empyema
7	18 yo	Intracranial abscess/ fluid collection	CSF	*Fusobacterium naviforme/nucleatum*	- Meningitis encephalitis panel, Gram stain and bacterial cultures negative, all specimens.	Intracranial abscess
8	13 yo	Meningoencephalitis	CSF<br>CSF	*Streptococcus intermedius*<br>*S. intermedius*	- Gram stains were Gram-positive cocci from same specimens.	Meningoencephalitis

No.	Age	Suspected clinical syndrome	Specimen source	16S rRNA gene PCR/sequencing test results	Conventional tests from same specimen source	Clinical diagnosis
					- Bacterial cultures grew *S. intermedius* from same specimens.	
9	5 do	Meningoencephalitis	CSF	*Staphylococcus epidermidis*	- Meningitis encephalitis panel and Gram stains negative, all specimens.<br>- Bacterial culture grew *S. epidermidis* from same specimen.	While this result most likely represents a skin contaminant, in the setting of a critically ill extremely premature infant this could be a pathogen, and we decided to treat it.
10	10 yo	Meningoencephalitis	CSF	*Streptococcus mitis* group, *Neisseria flavescens/perflava/subflava*	- Gram stains showed Gram-positive cocci in same specimens.<br>- Bacterial cultures grew *S. mitis* group from same specimens.	Meningoencephalitis in the setting of external ventricular drain
			Peri-implant tissue	*S. mitis* group, *N. flavescens/perflava/subflava*		
11	14 yo	Osteomyelitis	Synovial fluid	*S. aureus* complex	- Gram stains were negative from all specimens.<br>- Bacterial cultures grew *S. aureus* complex from four specimens.	Osteomyelitis
12	2 yo	Osteomyelitis	Bone	*S. aureus* complex, *Streptococcus agalactiae*	- Gram stain and bacterial cultures negative, all specimens.	Osteomyelitis
13	5 yo	Pleural effusion	Pleural fluid	*S. mitis* group	- Gram stain showed Gram-positive cocci resembling *Streptococcus* from same specimen.<br>- Bacterial cultures negative, all specimens.	Empyema
14	18 yo	Pleural effusion	Pleural fluid	*Fusobacterium necrophorum*	- Gram stains negative, all specimens. - Bacterial culture grew *Staphylococcus capitis* from a different specimen.	Empyema
15	5 yo	Pleural effusion	Pleural fluid	*S. mitis* group	- Gram stain and bacterial cultures negative, all specimens.	Empyema
16	1 yo	Pleural effusion	Pleural fluid	*S. mitis* group, most closely related to *Streptococcus pneumoniae*	- Gram stain showed Gram-positive cocci in same specimen. - Bacterial culture grew *S. pneumoniae* from same specimen.	Empyema
17	6 yo	Pleural effusion	Pleural fluid	*Streptococcus pyogenes*	- Gram stain and bacterial cultures negative, all specimens.	Empyema
18	1 yo	Septic arthritis	Synovial fluid	*Kingella kingae*	- Gram stain and bacterial cultures negative, all specimens.<br>- *K. kingae* PCR positive from same specimens.	Septic arthritis
			Synovial fluid	*K. kingae*		
			Synovial fluid	*K. kingae*		
19	2yo	Septic arthritis	Synovial fluid	*Fusobacterium naviforme/nucleatum*	- Gram stain and bacterial cultures negative, all specimens.	Unusual septic arthritis
20	15 yo	Septic arthritis	Synovial fluid	*Streptococcus dysgalactiae*	- Gram stains negative, all specimens.<br>- Bacterial culture grew one colony *S. dysgalactiae* from same specimen.	Septic arthritis

No.	Age	Suspected clinical syndrome	Specimen source	16S rRNA gene PCR/sequencing test results	Conventional tests from same specimen source	Clinical diagnosis
21	3 yo	Septic arthritis	Synovial fluid	*S. aureus* complex	- Gram stains negative, all specimens.<br>- Bacterial culture grew *S. aureus* from same specimen.	Septic arthritis
22	10 yo	Septic arthritis	Synovial fluid	*S. aureus* complex	- Gram stain showed Gram-positive cocci in same specimen.<br>- Bacterial culture grew *S. aureus* from same specimen.	Septic arthritis
23	6 yo	Traumatic wound infection	Vitreous fluid	*Pasteurella multocida, Capnocytophaga canimorsus, Enterobacter asburiae, Lactococcus* species	- Gram stain and bacterial cultures negative, all specimens.	Polymicrobial infection resulting from cat claw njury through the eyeball.
24	12 yo	Traumatic wound infection	Bone	*Bacillus* species*, Acinetobacter baumannii/calcoaceticus* complex, *Metamycoplasma hominis, Aerococcus* species*, Stenotrophomonas maltophilia, Flavobacteriaceae*	- Gram stain and bacterial cultures negative, all specimens.	Considered a contaminated wound with an open fracture following a motor vehicle accident.
			Bone	*Bacillus* species *A. baumannii/calcoaceticus* complex, *M. hominis, Aerococcus* species *S. maltophilia, Flavobacteriaceae, Enterobacteriaceae, Achromobacter* species		
			Synovial tissue	*Bacillus* species *A. baumannii/calcoaceticus* complex*, Aerococcus* species*, S. maltophilia, Corynebacterium* species		
			Bone	*Bacillus* species*, A. baumannii/calcoaceticus* complex, *M. hominis, Aerococcus* species*, S. maltophilia, Flavobacteriaceae, Enterobacteriaceae*		

*yo, year-old; mo, month-old; do, day-old; CSF, cerebrospinal fluid.
